# Centralized Fusion Approach to the Estimation Problem with Multi-Packet Processing under Uncertainty in Outputs and Transmissions

**DOI:** 10.3390/s18082697

**Published:** 2018-08-16

**Authors:** Raquel Caballero-Águila, Aurora Hermoso-Carazo, Josefa Linares-Pérez

**Affiliations:** 1 Departamento de Estadística, Universidad de Jaén, Paraje Las Lagunillas, 23071 Jaén, Spain; 2 Departamento de Estadística, Universidad de Granada, Avda. Fuentenueva, 18071 Granada, Spain; ahermoso@ugr.es (A.H.-C.); jlinares@ugr.es (J.L.-P.)

**Keywords:** least-squares filtering, least-squares smoothing, networked systems, random parameter matrices, random delays, packet dropouts

## Abstract

This paper is concerned with the least-squares linear centralized estimation problem in multi-sensor network systems from measured outputs with uncertainties modeled by random parameter matrices. These measurements are transmitted to a central processor over different communication channels, and owing to the unreliability of the network, random one-step delays and packet dropouts are assumed to occur during the transmissions. In order to avoid network congestion, at each sampling time, each sensor’s data packet is transmitted just once, but due to the uncertainty of the transmissions, the processing center may receive either one packet, two packets, or nothing. Different white sequences of Bernoulli random variables are introduced to describe the observations used to update the estimators at each sampling time. To address the centralized estimation problem, augmented observation vectors are defined by accumulating the raw measurements from the different sensors, and when the current measurement of a sensor does not arrive on time, the corresponding component of the augmented measured output predictor is used as compensation in the estimator design. Through an innovation approach, centralized fusion estimators, including predictors, filters, and smoothers are obtained by recursive algorithms without requiring the signal evolution model. A numerical example is presented to show how uncertain systems with state-dependent multiplicative noise can be covered by the proposed model and how the estimation accuracy is influenced by both sensor uncertainties and transmission failures.

## 1. Introduction

### 1.1. Background and Motivation

With the active development of computer and communication technologies, the estimation problem in multi-sensor network stochastic systems has become an important research topic in the last few years. The significant advantages of multi-sensor systems in practical situations (low cost, remote operation, simple installation, and maintenance) are obvious, and have triggered wide use of these systems in many areas, such as target tracking, communications, the manufacturing industry, etc. Moreover, they usually provide more information than traditional communication systems with a single sensor alone. In spite of these advantages, a sensor network is not generally a reliable communication medium, and together with the communication capacity limitations (network bandwidths or service capabilities, among others), may yield different uncertainties during data transmission, such as missing measurements, random delays, and packet dropouts.

The development of sensor networks motivates the necessity to desig fusion estimation algorithms which integrate the information from the different sensors and take these network-induced uncertainties into account to achieve a satisfactory performance. Depending on the way the information fusion is performed, there are two fundamental fusion techniques: the centralized fusion approach, in which the measurements from all sensors are sent to a central processor where the fusion is performed, and the distributed fusion approach, in which the measurements from each sensor are processed independently to obtain local estimators before being sent to the fusion center. The survey papers [1,2,3] can be examined for a wide view of these and other multi-sensor data fusion techniques.

As already indicated, centralized fusion architecture is based on a fusion centre that is able to receive, fuse, and process the data coming from every sensor; hence, centralized fusion estimation algorithms provide optimal signal estimators based on the measured outputs from all sensors and, consequently, when all of the sensors work correctly and the connections are perfect, they have the optimal estimation accuracy. In light of these concerns, it is not surprising that the study of the centralized and distributed fusion estimation problems in multi-sensor systems with network-induced uncertainties (in both the sensor measured outputs and the data transmission) has become an active research area in recent years. The estimation problem in systems with uncertainties in the sensor outputs (such as missing measurements, stochastic sensor gain degradation and fading measurements) is addressed in refs. [4,5,6], among others. In refs. [7,8,9,10], systems with failures during transmission (such as uncertain observations, random delays, and packet dropouts) are considered. Also, recent advances in the estimation, filtering, and fusion of networked systems with network-induced phenomena can be reviewed in refs. [11,12], where detailed overviews on this field are presented.

Since our aim in this paper is the design of centralized fusion estimators in multi-sensor network systems with measurements perturbed by random parameter matrices subject to random transmission failures (delays and packet dropouts), and multi-packet processing is considered, we discuss the research status of the estimation problem in networked systems with some of these characteristics.

### 1.2. Multi-Sensor Measured Outputs with Random Parameter Matrices

It is well known that in sensor-network environments, the measured outputs can be subject not only to additive noises, but also to multiplicative noise uncertainties due to several reasons, such as the presence of an intermittent sensor or hardware failure, natural or human-made interference, etc. For example, measurement equations that model the above-mentioned situations involving degradation of the sensor gain, or missing or fading measurements must include multiplicative noises described by random variables with values of [0,1]. So, random measurement parameter matrices provide a unified framework to address different simultaneous network-induced phenomena, and networked systems with random parameter matrices are used in different areas of science (see, e.g., refs. [13,14]). Also, systems with random sensor delays and/or multiple packet dropouts are transformed into equivalent observation models with random measurement matrices (see, e.g., ref. [15]). Hence, the estimation problem for systems with random parameter matrices has experienced increasing interest due to its diverse applications, and many estimation algorithms for such systems have been proposed over the last few years (see, e.g., refs. [16,17,18,19,20,21,22,23,24], and references therein).

### 1.3. Transmission Random Delays and Losses: Observation Predictor Compensation

Random delays and packet dropouts in the measurement transmissions are usually unavoidable and clearly deteriorate the performance of the estimators. For this reason, much effort has been made towards the study of the estimation problem to incorporate the effects of these transmission uncertainties, and several modifications of the standard estimation algorithms have been proposed (see, e.g., refs. [25,26,27], and references therein). In the estimation problem from measurements subject to transmission losses, when a packet drops out, the processor does not recieve a valid measurement and the most common compensation procedure is the hold-input mechanism which consists of processing the last measurement that was successfully transmitted. Unlike the approach to deal with losses, in ref. [28] the estimator of the lost measurement based on the information received previously is proposed as the compensator; this methodology significantly improves the estimations, since in cases of loss, all the previously received measurements are considered, instead of using only the last one. In view of this clear improvement of the estimators, the compensation strategy developed in ref. [28] has been adopted in some other recent investigations (see, e.g., refs. [29,30], and references therein).

### 1.4. Multi-Packet Processing

Another concern at the forefront of research in networked systems subject to random delays and packet dropouts is the number of packets that are processed to update the estimator at each moment, and different observation models have been proposed to deal with this issue. For example, to avoid losses as much as possible, in ref. [16] it is assumed that each packet is transmitted several times. In contrast, to avoid the network congestion that may be caused by multiple transmissions, ref. [31] the packets are sent just once. These papers also assume that each packet is either received on time, delayed for, at most, one sampling time, or lost, and only one packet or no packets are processed to update the estimator at each moment. However, in refs. [32,33,34] two packets were able to arrive at each sampling time, in which case, both were used to update the estimators, thus improving their performance. In these papers, different packet dropout compensation procedures have been employed. The last available measurement was used as compensation in refs. [32,34], while the observation predictor was considered in ref. [34].

### 1.5. Addressed Problem and Paper Contributions

Based on the considerations made above, we were motivated to address the study of the centralized fusion estimation problem for multi-sensor networked systems perturbed by random parameter matrices. This problem is discussed under the following assumptions: (*a*) Each sensor transmits their measured outputs to a central processor over different communication channels and random delays, and packet dropouts are assumed to occur during the transmission; (*b*) in order to avoid the network congestion, at each time instant, the different sensors send their packets only once, but due to the transmission random failures, the processing center can receive more than one packet; specifically, either one packet, two packets, or nothing; and (*c*) the measurement output predictor is used as a loss compensation strategy.

The main contributions and advantages of the current work are summarized as follows: (*1*) A unified framework to deal with different network-induced phenomena in the measured outputs, such as missing measurements or sensor gain degradation, is provided by the use of random measurement matrices. (*2*) Besides the uncertainties in the measured outputs, random one-step delays and packet dropouts are assumed to occur during the transmission at different rates at each sensor. Unlike previous authors’ papers concerning random measurement matrices and random transmission delays and losses where only one packet is processed to update the estimator at each moment, in this paper, the estimation algorithms are obtained under the assumption that either one packet, two packets, or nothing may arrive at each sampling time. (*3*) Concerning the compensation strategy, the use of the measurement predictor as the loss compensator combined with the simultaneous processing of delayed packets provides better estimators in comparison to other approaches where the last measurement successfully received is used to compensate the data packets and only one packet is processed to update the estimator at each moment. (*4*) The centralized fusion estimation problem is addressed using covariance information, without requiring full knowledge of the state-space model generating the signal process, thus providing a general approach to deal with different kinds of signal processes. (*5*) The innovation approach is used to obtain recursive prediction, filtering, and fixed-point smoothing algorithms which are recursive and computationally simple, and thus aresuitable for online implementation. In contrast to the approaches where the state augmentation technique is used, the proposed algorithms are deduced without making use of augmented systems; therefore, they have lower computational costs than those based on the augmentation method.

### 1.6. Paper Structure and Notation

The remaining sections of the paper are organized as follows. Section 2 presents the assumptions for the signal process, the mathematical models of the multi-sensor measured outputs with random parameter matrices, and the measurements received by the central processor with random delays and packet losses. Section 3 provides the main results of the research, namely, the covariance-based centralized least-squares linear prediction and filtering algorithm (Theorem 1) and fixed-point smoothing algorithm (Theorem 2). A numerical example is presented in Section 4 to show the performance of the proposed centralized estimators, and some concluding remarks are drawn in Section 5. The proofs of Theorems 1 and 2 are presented in the Appendix A and Appendix B, respectively.

The notations used throughout the paper are standard. $R^{n}$ and $R^{m\times n}$ denote the *n*-dimensional Euclidean space and the set of all m×n real matrices, respectively. $A^{T}$ and $A^{-1}$ denote the transpose and inverse of a matrix (*A*), respectively. $I_{n}$ and $0_{n}$ denote the n×n identity matrix and zero matrix, respectively. $1_{n}$ denotes the all-ones vector. Finally, ⊗ and ∘ are the Kronecker and Hadamard products, respectively, and $\delta_{k,s}$ is the Kronecker delta function.

## 2. Observation Model and Preliminaries

The aim of this section is to design a mathematical model to allow the observations to be processed in the least-squares (LS) linear estimation problem of discrete-time signal processes from multi-sensor noisy measurements transmitted through imperfect communication channels where random one-step delay and packet dropouts may arise in the transmission process. More specifically, in order to avoid the network congestion, at every sampling time, it is assumed that the measured outputs from each sensor, which are perturbed by random parameter matrices, are transmitted just once to a central processor, and due to random delays and losses, the processing center (PC) may receive, from each sensor, either one packet, two packets, or nothing at each time instant.

In this context, our goal is to find recursive algorithms for the LS linear prediction, filtering, and fixed-point smoothing problems using the centralized fusion method. We assume that only information about the mean and covariance functions of the signal process is available, and this information is specified in the following hypothesis:

**(H1)**  The $n_{x}$-dimensional signal process, ${\left\{x_{k}\right\}}_{k\geq 1}$, has a zero-mean, and its autocovariance function is expressed in a separable form, $E\left[x_{k}x_{s}^{T}\right]=A_{k}B_{s}^{T}$, s≤k, where $A_{k},B_{s}\in R^{n_{x}\times M}$ are known matrices.

### 2.1. Multi-Sensor Measured Outputs with Random Parameter Matrices

We assume that there are *m* sensors which provide measured outputs of the signal process that are affected by random parameter matrices according to the following model:

(1) $$z_{k}^{\left(i\right)}=H_{k}^{\left(i\right)}x_{k}+v_{k}^{\left(i\right)},\;\;\;\;k\geq 1,\;\;\;\;\;i=1,\ldots ,m,$$

where $z_{k}^{\left(i\right)}\in R^{n_{z}}$ is the signal measurement in the *i*-th sensor at time *k*, $H_{k}^{\left(i\right)}$ are random parameter matrices, and $v_{k}^{\left(i\right)}$ are the measurement noises. We assume the following hypotheses for these proceses:

**(H2)**  ${\left\{H_{k}^{\left(i\right)}\right\}}_{k\geq 1}$, for i=1,…,m, are independent sequences of independent random parameter matrices. For $p=1,\ldots ,n_{z}$ and $q=1,\ldots ,n_{x}$, we denote $h_{{}_{pq}}^{\left(i\right)}\left(k\right)$ as the (p,q)-th entry of $H_{k}^{\left(i\right)}$, which has known first and second order moments, and ${\bar{H}}_{k}^{\left(i\right)}=E\left[H_{k}^{\left(i\right)}\right]$.
**(H3)**  The measurement noises ${\left\{v_{k}^{\left(i\right)}\right\}}_{k\geq 1}$, i=1,…,m, are zero-mean second-order white processes with $E\left[v_{k}^{\left(i\right)}v_{s}^{(j)T}\right]=R_{k}^{\left(ij\right)}\delta_{k,s}$.

### 2.2. Observation Model. Properties

To address the estimation problem with the centralized fusion method, the observations coming from the different sensors are gathered and jointly processed at each sampling time to yield the optimal signal estimator. So, the problem is addressed by considering, at each time (k≥1), the vector constituted by the measurements received from all sensors and for this purpose, Equations (1) were combined to yield the following stacked measured output equation:

(2) $$\begin{array}{c} z_{k}=H_{k}x_{k}+v_{k},\;\;k\geq 1, \end{array}$$

where $z_{k}={\left(z_{k}^{(1)T},\ldots ,z_{k}^{(m)T}\right)}^{T}\;\;,\;\;H_{k}={\left(H_{k}^{(1)T}\;\;,\ldots ,H_{k}^{(m)T}\right)}^{T}\;\;,\;\;v_{k}={\left(v_{k}^{(1)T},\ldots ,v_{k}^{(m)T}\right)}^{T}\;.$

As already indicated, random one-step delays and packet dropouts occur during the transmissions to the PC. To model these failures, we introduced the following sequences of random variables:

- ${\left\{\gamma_{k}^{\left(i\right)}\right\}}_{k\geq 1},\;i=1,\ldots ,m$, are sequences of Bernoulli random variables. Each i=1,…,m, $\gamma_{k}^{\left(i\right)}=0$ means that the output at the current sampling time, $z_{k}^{\left(i\right)}$, arrives on time to be processed for the estimation, while $\gamma_{k}^{\left(i\right)}=1$ means that this output is either delayed or dropped out; and
- ${\left\{\psi_{k}^{\left(i\right)}\right\}}_{k\geq 2},\;i=1,\ldots ,m$, are sequences of Bernoulli random variables. For each i=1,…,m, $\psi_{k}^{\left(i\right)}=1$ means that $z_{k-1}^{\left(i\right)}$ is processed at sampling time *k* (because it was one-step delayed) and $\psi_{k}^{\left(i\right)}=0$ means that $z_{k-1}^{\left(i\right)}$ is not processed at sampling time *k* (because it was either received at time k−1 or dropped out). Since $\gamma_{k-1}^{\left(i\right)}=0$ implies $\psi_{k}^{\left(i\right)}=0$, it is clear that the value of $\psi_{k}^{\left(i\right)}$ is conditioned by that of $\gamma_{k-1}^{\left(i\right)}$.

For the previous sequences of Bernoulli variables, we assume the following hypothesis:

**(H4)**  ${\left\{{\left(\gamma_{k}^{\left(i\right)},\psi_{k+1}^{\left(i\right)}\right)}^{T}\right\}}_{k\geq 1},\;i=1,\ldots ,m$, are independent sequences of independent random vectors, such that
  - ${\left\{\gamma_{k}^{\left(i\right)}\right\}}_{k\geq 1},\;\;i=1,\ldots ,m$, are sequences of Bernoulli random variables with known probabilities, $P\left(\gamma_{k}^{\left(i\right)}=1\right)={\bar{\gamma }}_{k}^{\left(i\right)}$, k≥1; and
  - ${\left\{\psi_{k}^{\left(i\right)}\right\}}_{k\geq 2},\;\;i=1,\ldots ,m$, are sequences of Bernoulli random variables such that the conditional probabilities ($P(\psi_{k}^{\left(i\right)}=1/\gamma_{k-1}^{\left(i\right)}=1)$) are known. Thus,
    $${\bar{\psi }}_{k}^{\left(i\right)}\equiv P\left(\psi_{k}^{\left(i\right)}=1\right)=P\left(\psi_{k}^{\left(i\right)}=1/\gamma_{k-1}^{\left(i\right)}=1\right){\bar{\gamma }}_{k-1}^{\left(i\right)},\;\;k\geq 2.$$

Moreover, the mutual independence hypothesis of the involved processes is also necessary:

**(H5)**  For i=1,…,m, the signal process ${\left\{x_{k}\right\}}_{k\geq 1}$, the random matrices ${\left\{H_{k}^{\left(i\right)}\right\}}_{k\geq 1}$, and the noises ${\left\{v_{k}^{\left(i\right)}\right\}}_{k\geq 1}$ and ${\left\{{\left(\gamma_{k}^{\left(i\right)},\psi_{k+1}^{\left(i\right)}\right)}^{T}\right\}}_{k\geq 1}$ are mutually independent.

**Remark** **1.** *From hypothesis (H4), for i,j=1,…,m, the following correlations are clear:*

(3) $$\begin{array}{c} E\left[\gamma_{k}^{\left(i\right)}\gamma_{k}^{\left(j\right)}\right]=\left\{\begin{array}{c} {\bar{\gamma }}_{k}^{\left(i\right)},\;\;i=j,\;\\ {\bar{\gamma }}_{k}^{\left(i\right)}{\bar{\gamma }}_{k}^{\left(j\right)},\;\;i\neq j. \end{array}\right.\;\;\;\;E\left[\psi_{k}^{\left(i\right)}\psi_{k}^{\left(j\right)}\right]=\left\{\begin{array}{c} {\bar{\psi }}_{k}^{\left(i\right)},\;\;i=j,\;\\ {\bar{\psi }}_{k}^{\left(i\right)}{\bar{\psi }}_{s}^{\left(j\right)},\;\;i\neq j. \end{array}\right.\\ E\left[\psi_{k+1}^{\left(i\right)}\left(1-\gamma_{k}^{\left(j\right)}\right)\right]=\left\{\begin{array}{c} 0,\;\;i=j,\;\\ {\bar{\psi }}_{k+1}^{\left(i\right)}\left(1-{\bar{\gamma }}_{k}^{\left(j\right)}\right),\;\;i\neq j. \end{array}\right. \end{array}$$

In order to write jointly the sensor measurements to be processed at each sampling time, we defined the matrices $\Gamma_{k}\equiv Diag\left(\gamma_{k}^{\left(1\right)},\ldots ,\gamma_{k}^{\left(m\right)}\right)⊗I_{n_{z}}$. and $\Psi_{k}\equiv Diag\left(\psi_{k}^{\left(1\right)},\ldots ,\psi_{k}^{\left(m\right)}\right)⊗I_{n_{z}},\;k\geq 1$. From the definition of variables $\gamma_{k}^{\left(i\right)},\;i=1,\ldots ,m,$, it is clear that the non-zero components of vector $\left(I_{mn_{z}}-\Gamma_{k}\right)z_{k}$ are those of $z_{k}$ that arrive on time at the PC and, consequently, those processed at time *k*. The other components of $z_{k}$ are delayed or lost, and as compensation, the corresponding components of the predictor ${\hat{z}}_{k/k-1}$, specified in $\Gamma_{k}{\hat{z}}_{k/k-1}$, are processed. Similarly, the non-zero components of $\Psi_{k}z_{k-1}$ are those of $z_{k-1}$ that are affected by one-step delay, and consequently, they are also processed at time *k*. Hence, the processed observations at each time are expressed by the following model:

(4) $$y_{k}=\left(\begin{array}{c} \left(I_{mn_{z}}-\Gamma_{k}\right)z_{k}+\Gamma_{k}{\hat{z}}_{k/k-1}\\ \Psi_{k}z_{k-1} \end{array}\right),\;\;k\geq 2;\;\;\;\;\;\;\;\;\;y_{1}=\left(\begin{array}{c} \left(I_{mn_{z}}-\Gamma_{1}\right)z_{1}\\ 0 \end{array}\right),$$

or equivalently,

(5) $$\begin{array}{c} y_{k}=C_{0}\left(I_{mn_{z}}-\Gamma_{k}\right)z_{k}+C_{0}\Gamma_{k}{\hat{z}}_{k/k-1}+C_{1}\Psi_{k}z_{k-1},\;\;k\geq 2;\\ y_{1}=C_{0}\left(I_{mn_{z}}-\Gamma_{1}\right)z_{1}, \end{array}$$

where $C_{0}={\left(\;I_{mn_{z}}\;,\;0_{mn_{z}}\;\right)}^{T}$ and $C_{1}={\left(\;0_{mn_{z}}\;,\;I_{mn_{z}}\;\right)}^{T}.$

**Remark** **2.** *For a better understanding of Model (4) for the measurements processed after the possible transmission one-step delays and losses, a single sensor is considered in the following comments. On the one hand, note that $\gamma_{k}=0$ means that the output at the current sampling time ($z_{k}$) arrives on time to be processed. Then, if $\psi_{k}=1$, the measurement processed at time k is $y_{k}={\left(\begin{array}{cc} z_{k}^{T} & z_{k-1}^{T} \end{array}\right)}^{T}$, while if $\psi_{k}=0$, then $y_{k}={\left(\begin{array}{cc} z_{k}^{T} & 0 \end{array}\right)}^{T}$. On the other hand, if $\gamma_{k}=1$, the output $z_{k}$ is either delayed or dropped out, and its predictor ${\hat{z}}_{k/k-1}$ is processed at time k. Then, if $\psi_{k}=1$, the measurement processed at time k is $y_{k}={\left(\begin{array}{cc} {\hat{z}}_{k/k-1}^{T} & z_{k-1}^{T} \end{array}\right)}^{T}$, while if $\psi_{k}=0$, then $y_{k}={\left(\begin{array}{cc} {\hat{z}}_{k/k-1}^{T} & 0 \end{array}\right)}^{T}$. Table 1 displays ten iterations of a specific simulation of packet transmission.*

*From Table 1, it can be observed that $z_{1}$, $z_{3}$, $z_{6}$, $z_{7}$, and $z_{9}$ arrive on time to be processed; $z_{2}$, $z_{4}$ and $z_{8}$ are one-step delayed; and $z_{5}$ and $z_{10}$ are lost. So, Model (4) describes possible one-step random transmission delays and packet dropouts in networked systems, where one or two packets can be processed for the estimation. Finally, note that the predictors ${\hat{z}}_{k/k-1}$, k=2,4,5,8,10 are used to compensate for the measurements that do not arrive on time.*

The problem is then formulated as that of obtaining the LS linear estimator of the signal, $x_{k}$ based on the observations $\left\{y_{1},\ldots ,y_{L}\right\}$ given in (5). Next, some statistical properties of the processes involved in observation models (2) and (5), which are necessary to address the LS linear estimation problem, are specified:

**(P1)**  ${\left\{H_{k}\right\}}_{k\geq 1}$ is a sequence of independent random matrices with known means: ${\bar{H}}_{k}\equiv E\left[H_{k}\right]={\left({\bar{H}}_{k}^{(1)T},\ldots ,{\bar{H}}_{k}^{(m)T}\right)}^{T},\;\;k\geq 1$.
**(P2)**  The sequence ${\left\{v_{k}\right\}}_{k\geq 1}$ is a zero-mean second-order process with $E\left[v_{k}v_{s}^{T}\right]=R_{k}\delta_{k,s},$ where $R_{k}={\left(R_{k}^{\left(ij\right)}\right)}_{i,j=1,\ldots ,m}$.
**(P3)**  The random matrices ${\left\{\left(\Gamma_{k},\Psi_{k+1}\right)\right\}}_{k\geq 1}$ are independent, and their means are given by
  $${\bar{\Gamma }}_{k}\equiv E\left[\Gamma_{k}\right]=Diag\left({\bar{\gamma }}_{k}^{\left(1\right)},\ldots ,{\bar{\gamma }}_{k}^{\left(m\right)}\right)⊗I_{n_{z}},\;\;\;\;{\bar{\Psi }}_{k}\equiv E\left[\Psi_{k}\right]=Diag\left({\bar{\psi }}_{k}^{\left(1\right)},\ldots ,{\bar{\psi }}_{k}^{\left(m\right)}\right)⊗I_{n_{z}}.$$
**(P4)**  The signal process, ${\left\{x_{k}\right\}}_{k\geq 1}$ and the processes ${\left\{H_{k}\right\}}_{k\geq 1}$ ${\left\{v_{k}\right\}}_{k\geq 1}$ and ${\left\{\left(\Gamma_{k},\Psi_{k+1}\right)\right\}}_{k\geq 1}$ are mutually independent.
**(P5)**  ${\left\{z_{k}\right\}}_{k\geq 1}$ is a zero-mean process with covariance matrices $\Sigma_{k,s}^{z}\equiv E\left[z_{k}z_{s}^{T}\right]$, for s≤k which, from (P4), are given by
  $$\Sigma_{k,s}^{z}=E\left[H_{k}A_{k}B_{s}^{T}H_{s}^{T}\right]+R_{k}\delta_{k,s},\;s\leq k,$$
  with $E\left[H_{k}A_{k}B_{s}^{T}H_{s}^{T}\right]={\bar{H}}_{k}A_{k}B_{s}^{T}{\bar{H}}_{s}^{T}$, for s<k, and
  $$E\left[H_{k}A_{k}B_{k}^{T}H_{k}^{T}\right]={\left(E[H_{k}^{\left(i\right)}A_{k}B_{k}^{T}H_{k}^{(j)T}]\right)}_{i,j=1,\ldots ,m},$$
  where the (p,q)-th entries of the matrices $E[H_{k}^{\left(i\right)}A_{k}B_{k}^{T}H_{k}^{(j)T}]$ are given by
  $${\left(E[H_{k}^{\left(i\right)}A_{k}B_{k}^{T}H_{k}^{(j)T}]\right)}_{\;pq}\;=\;\sum_{a=1}^{n_{x}}\sum_{b=1}^{n_{x}}\;E\left[h_{{}_{pa}}^{\left(i\right)}\left(k\right)h_{{}_{qb}}^{\left(j\right)}\left(k\right)\right]\;{\left(A_{k}B_{k}^{T}\right)}_{ab},\;\;p,q=1,\ldots ,n_{z}.$$

**Remark** **3.** *By denoting $\gamma_{k}={\left(\gamma_{k}^{\left(1\right)},\ldots ,\gamma_{k}^{\left(m\right)}\right)}^{T}\;⊗1_{n_{z}}$ and $\psi_{k}={\left(\psi_{k}^{\left(1\right)},\ldots ,\psi_{k}^{\left(m\right)}\right)}^{T}⊗1_{n_{z}}$, it is clear that $K_{k}^{1-\gamma }\equiv E\left[\left(1_{mn_{z}}-\gamma_{k}\right){\left(1_{mn_{z}}-\gamma_{k}\right)}^{T}\right]$ and $K_{k}^{\psi }\equiv E\left[\psi_{k}\psi_{k}^{T}\right]$ are known matrices whose entries are given in (3). Now, by defining*

(6) $$\begin{array}{c} \xi_{k}=C_{0}\left(I_{mn_{z}}-\Gamma_{k}\right)z_{k}+C_{1}\Psi_{k}z_{k-1},\;\;k\geq 2;\;\;\;\;\xi_{1}=C_{0}\left(I_{mn_{z}}-\Gamma_{1}\right)z_{1}, \end{array}$$

*and taking the Hadamard product properties into account, it is easy to check that the covariance matrices ($\Sigma_{k}^{\xi }\equiv E\left[\xi_{k}\xi_{k}^{T}\right]$) are given by*

(7) $$\begin{array}{cc} \Sigma_{k}^{\xi } & =C_{0}\left(K_{k}^{1-\gamma }\circ \Sigma_{k}^{z}\right)C_{0}^{T}+C_{1}\left(K_{k}^{\psi }\circ \Sigma_{k-1}^{z}\right)C_{1}^{T}\\ & +C_{0}\left(I_{mn_{z}}-{\bar{\Gamma }}_{k}\right)\Sigma_{k,k-1}^{z}{\bar{\Psi }}_{k}C_{1}^{T}+C_{1}{\bar{\Psi }}_{k}\Sigma_{k,k-1}^{zT}\left(I_{mn_{z}}-{\bar{\Gamma }}_{k}\right)C_{0}^{T},\;\;\;k\geq 2;\\ \Sigma_{1}^{\xi } & =C_{0}\left(K_{1}^{1-\gamma }\circ \Sigma_{k}^{1}\right)C_{0}^{T}. \end{array}$$

## 3. Centralized Fusion Estimators

This section is concerned with the problem of obtaining recursive algorithms for the LS linear centralized fusion prediction, filtering, and fixed-point smoothing estimators. For this purpose, we used an innovation approach. Also the estimation error covariance matrices, which are used to measure the accuracy of the proposed estimators when the LS optimality criterion is used, were derived.

**Figure 1:**
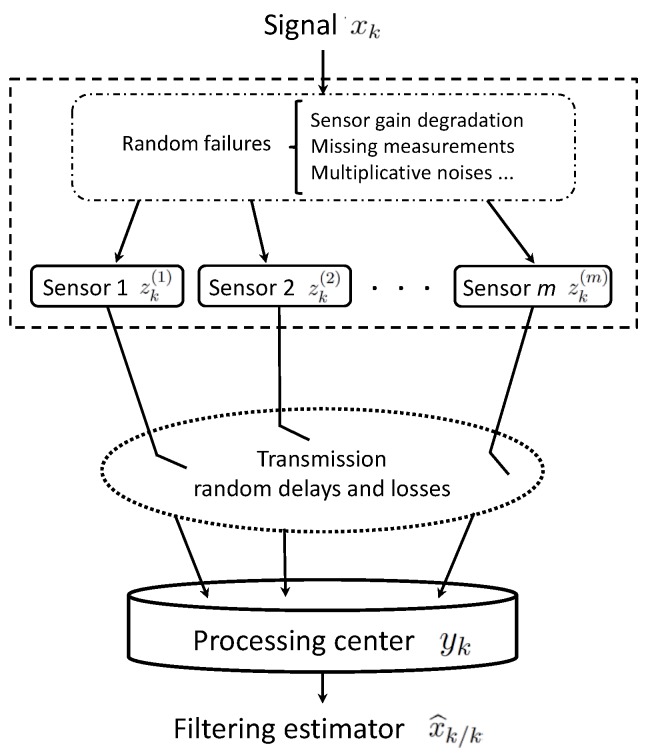
Centralized fusion filtering estimation with random uncertainties in measured outputs and transmission.

The centralized fusion structure for the considered networked systems with random uncertainties in the measured outputs and transmission is illustrated in Figure 1.

### 3.1. Innovation Technique

As indicated above, our aim was to obtain the optimal LS linear estimators, ${\hat{x}}_{k/L}$, of the signal $x_{k}$ based on the measurements $\left\{y_{1},\ldots ,y_{L}\right\}$, given in (5), by recursive algorithms. Since the estimator ${\hat{x}}_{k/L}$ is the orthogonal projection of the signal $x_{k}$ onto the linear space spanned by the nonorthogonal vectors $\left\{y_{1},\ldots ,y_{L}\right\}$, we used an innovation approach in which the observation process ${\left\{y_{k}\right\}}_{k\geq 1}$ was transformed into an equivalent one (*innovation process*) of orthogonal vectors ${\left\{\mu_{k}\right\}}_{k\geq 1}$; the equivalence means that each set $\left\{\mu_{1},\ldots ,\mu_{L}\right\}$ spans the same linear subspace as $\left\{y_{1},\ldots ,y_{L}\right\}$.

The innovation at time *k* is defined as $\mu_{k}=y_{k}-{\hat{y}}_{k/k-1}$, where ${\hat{y}}_{1/0}=E\left[y_{1}\right]=0$ and, for k≥2, ${\hat{y}}_{k/k-1}$, the one-stage linear predictor of $y_{k}$ is the projection of $y_{k}$ onto the linear space generated by $\left\{\mu_{1},\ldots ,\mu_{k-1}\right\}$. Due to the orthogonality property of the innovations and since the innovation process is uniquely determined by the observations, by replacing the observation process by the innovation one, the following general expression for the LS linear estimators of any vector $w_{k}$ based on the observations $\left\{y_{1},\ldots ,y_{L}\right\}$ was obtained

(8) $${\hat{w}}_{k/L}=\sum_{h=1}^{L}E\left[w_{k}\mu_{h}^{T}\right]{\left(E\left[\mu_{h}\mu_{h}^{T}\right]\right)}^{-1}\mu_{h}.$$

This expression is derived from the uncorrelation property of the estimation error with all of the innovations, which is guaranteed by the Orthogonal Projection Lemma (OPL). As shown by (8), the first step to obtain the signal estimators is to find an explicit formula for the innovation or, equivalently, for the one-stage linear predictor of the observation.

#### One-Stage Observation Predictor

To obtain ${\hat{y}}_{k/k-1}$, the projection of $y_{k}$ onto the linear space generated by $\left\{\mu_{1},\ldots ,\mu_{k-1}\right\}$, we used (5) and we note that $\Psi_{k}$ and $H_{k-1}$ are correlated with the innovation $\mu_{k-1}$. So, to simplify the derivation of ${\hat{y}}_{k/k-1}$, the observations (5) were rewritten as follows:

(9) $$\begin{array}{c} y_{k}=C_{0}\left(I_{mn_{z}}\;-\Gamma_{k}\right)z_{k}+C_{1}{\bar{\Psi }}_{k}{\bar{H}}_{k-1}x_{k-1}+C_{0}\Gamma_{k}{\hat{z}}_{k/k-1}+V_{k-1},\;\;k\geq 2,\\ V_{k}=C_{1}\Psi_{k+1}z_{k}-C_{1}{\bar{\Psi }}_{k+1}{\bar{H}}_{k}x_{k},\;\;k\geq 1. \end{array}$$

Taking into account that $\Psi_{k+1}$ and $H_{k}$ are independent of $\mu_{h}$, for h≤k−1, it is easy to see that $E\left[V_{k}\mu_{h}^{T}\right]=0$ for h≤k−1. So, from the general expression (8), we obtained ${\hat{V}}_{k/k}=V_{k}\Pi_{k}^{-1}\mu_{k},\;\;k\geq 1$, where $V_{k}\equiv E\left[V_{k}\mu_{k}^{T}\right]=E\left[V_{k}y_{k}^{T}\right]$. Hence, according to the projection theory, ${\hat{y}}_{k/k-1}$ satisfies

(10) $$\begin{array}{c} {\hat{y}}_{k/k-1}=C_{0}{\bar{H}}_{k}{\hat{x}}_{k/k-1}+C_{1}{\bar{\Psi }}_{k}{\bar{H}}_{k-1}{\hat{x}}_{k-1/k-1}+V_{k-1}\Pi_{k-1}^{-1}\mu_{k-1},\;\;k\geq 2. \end{array}$$

This expression for the one-stage observation predictor along with (8) for the LS linear estimators are the starting points to get the recursive prediction, filtering, and fixed-point smoothing algorithms.

### 3.2. Centralized Fusion Prediction, Filtering, and Smoothing Algorithms

The following theorem presents a recursive algorithm for the LS linear centralized fusion prediction and filtering estimators ${\hat{x}}_{k/L}$, L≤k, of the signal $x_{k}$ based on the observations $\left\{y_{1},\ldots ,y_{L}\right\}$ given in (5) or equivalently, in (9).

**Theorem** **1.** *Under hypotheses (H1)–(H5), the LS linear centralized predictors and filter ${\hat{x}}_{k/L}$, L≤k and the corresponding error covariance matrices ${\hat{\Sigma }}_{k/L}\equiv E\left[\left(x_{k}-{\hat{x}}_{k/L}\right)\left(x_{k}-{\hat{x}}_{k/L}^{T}\right)\right]$ are obtained by*

(11) $${\hat{x}}_{k/L}=A_{k}e_{L},\;\;\;\;\;\;{\hat{\Sigma }}_{k/L}=A_{k}{\left(B_{k}-A_{k}\Sigma_{L}^{e}\right)}^{T},\;\;L\leq k,$$

*where the vectors $e_{L}$ and the matrices $\Sigma_{L}^{e}\equiv E\left[e_{L}e_{L}^{T}\right]$ are recursively obtained from*

(12) $$e_{L}=e_{L-1}+E_{L}\Pi_{L}^{-1}\mu_{L},\;\;L\geq 1;\;\;\;e_{0}=0,$$

(13) $$\Sigma_{L}^{e}=\Sigma_{L-1}^{e}+E_{L}\Pi_{L}^{-1}E_{L}^{T},\;\;L\geq 1;\;\;\Sigma_{0}^{e}=0,$$

*and the matrices $E_{L}\equiv E\left[e_{L}\mu_{L}^{T}\right]$ satisfy*

(14) $$E_{L}={\bar{H}}_{B_{L}}^{T}-\Sigma_{L-1}^{e}{\bar{H}}_{A_{L}}^{T}-E_{L-1}\Pi_{L-1}^{-1}V_{L-1}^{T},\;\;L\geq 2;\;\;\;E_{1}={\bar{H}}_{B_{1}}^{T},$$

*where ${\bar{H}}_{D_{L}}$, for D=A,B, is defined by*

(15) $$\begin{array}{c} {\bar{H}}_{D_{L}}=C_{0}\left(I_{mn_{z}}-{\bar{\Gamma }}_{L}\right){\bar{H}}_{L}D_{L}+C_{1}{\bar{\Psi }}_{L}{\bar{H}}_{L-1}D_{L-1},\;\;L\geq 2;\;\;{\bar{H}}_{D_{1}}=C_{0}\left(I_{mn_{z}}-{\bar{\Gamma }}_{1}\right){\bar{H}}_{1}D_{1}. \end{array}$$

*The innovations $\mu_{L}=y_{L}-{\hat{y}}_{L/L-1}$ are given by*

(16) $$\begin{array}{c} \mu_{L}=y_{L}-\left({\bar{H}}_{A_{L}}+C_{0}{\bar{\Gamma }}_{L}{\bar{H}}_{L}A_{L}\right)e_{L-1}-V_{L-1}\Pi_{L-1}^{-1}\mu_{L-1},\;\;L\geq 2;\;\;\;\mu_{1}=y_{1}, \end{array}$$

*and the coefficients $V_{L}=E\left[V_{L}\mu_{L}^{T}\right],$ are obtained by*

(17) $$\begin{array}{c} V_{L}=C_{1}(K_{L+1,L}^{\psi (1-\gamma )}\circ \left(\Sigma_{L}^{z}-{\bar{H}}_{L}A_{L}\Sigma_{L-1}^{e}A_{L}^{T}{\bar{H}}_{L}^{T}\right)\\ \;-{\bar{\Psi }}_{L+1}{\bar{H}}_{L}A_{L}{\left(B_{L}-A_{L}\Sigma_{L-1}^{e}\right)}^{T}{\bar{H}}_{L}^{T}\left(I-{\bar{\Gamma }}_{L}\right))C_{0}^{T},\;\;L\geq 1, \end{array}$$

*where $K_{L+1,L}^{\psi (1-\gamma )}\equiv E\left[\psi_{L+1}{\left(1_{mn_{z}}-\gamma_{L}\right)}^{T}\right]$, whose entries are given in (3).*

*The innovation covariance matrices $\Pi_{L}\equiv E\left[\mu_{L}\mu_{L}^{T}\right]$ satisfy*

(18) $$\begin{array}{cc} \Pi_{L} & =\Sigma_{L}^{\xi^{}}-C_{0}\left[K_{L}^{\gamma }\circ \left({\bar{H}}_{L}A_{L}\Sigma_{L-1}^{e}A_{L}^{T}{\bar{H}}_{L}^{T}\right)\right]C_{0}^{T}+O_{L,L-1}A_{L}^{T}{\bar{H}}_{L}^{T}{\bar{\Gamma }}_{L}C_{0}^{T}\\ & -{\bar{H}}_{A_{L}}^{}O_{L,L-1}^{T}-V_{L-1}^{}\Pi_{L-1}^{-1}Y_{L,L-1}^{T},\;L\geq 2;\;\;\;\;\Pi_{1}=\Sigma_{1}^{\xi }, \end{array}$$

*where the matrices $\Sigma_{L}^{\xi }$ are given in (7), $K_{L}^{\gamma }\equiv E\left[\gamma_{L}\gamma_{L}^{T}\right]$, whose entries are obtained by (3), and the matrices $O_{L,L-1}\equiv E\left[y_{L}e_{L-1}^{T}\right]$ and $Y_{L,L-1}\equiv E\left[y_{L}\mu_{L-1}^{T}\right]$ are given by*

(19) $$\begin{array}{c} O_{L,L-1}=\left({\bar{H}}_{A_{L}}+C_{0}{\bar{\Gamma }}_{L}{\bar{H}}_{L}A_{L}\right)\Sigma_{L-1}^{e}+V_{L-1}\Pi_{L-1}^{-1}E_{L-1}^{T},\;\;L\geq 2.\\ Y_{L,L-1}=\left({\bar{H}}_{A_{L}}+C_{0}{\bar{\Gamma }}_{L}{\bar{H}}_{L}A_{L}\right)E_{L-1}+V_{L-1},\;\;L\geq 2. \end{array}$$

**Proof.**  See Appendix A. ☐

Next, a recursive algorithm for the LS linear centralized fusion smoothers ${\hat{x}}_{k/k+h}$ at the fixed point *k* for any h≥1 is established in the following theorem.

**Theorem** **2.** *Under hypotheses (H1)–(H5), the LS linear centralized fixed-point smoothers ${\hat{x}}_{k/k+h}$ are calculated by*

(20) $${\hat{x}}_{k/k+h}={\hat{x}}_{k/k+h-1}+X_{k,k+h}\Pi_{k+h}^{-1}\mu_{k+h},\;\;k\geq 1,\;\;\;h\geq 1,$$

*whose initial condition is given by the centralized filter ${\hat{x}}_{k/k}$, and the matrices $X_{k,k+h}=E\left[x_{k}\mu_{k+h}^{T}\right]$ are obtained by*

(21) $$\begin{array}{c} X_{k,k+h}=\left(B_{k}-E_{k,k+h-1}\right){\bar{H}}_{A_{k+h}}^{T}-X_{k,k+h-1}\Pi_{k+h-1}^{-1}V_{k+h-1}^{T},\;\;h\geq 1;\\ X_{k,k}=A_{k}E_{k}. \end{array}$$

*The matrices $E_{k,k+h}=E\left[x_{k}e_{k+h}^{T}\right]$ satisfy the following recursive formula:*

(22) $$E_{k,k+h}=E_{k,k+h-1}+X_{k,k+h}\Pi_{k+h}^{-1}E_{k+h}^{T},\;\;h\geq 1;\;\;\;E_{k,k}=A_{k}\Sigma_{k}^{e}.$$

*The fixed-point smoothing error covariance matrices, ${\hat{\Sigma }}_{k/k+h}\equiv E\left[\left(x_{k}-{\hat{x}}_{k/k+h}\right){\left(x_{k}-{\hat{x}}_{k/k+h}\right)}^{T}\right]$, are calculated by*

$${\hat{\Sigma }}_{k/k+h}={\hat{\Sigma }}_{k/k+h-1}-X_{k,k+h}\Pi_{k+h}^{-1}X_{k,k+h}^{T},\;\;k\geq 1,\;\;\;h\geq 1,$$

*with the initial condition given by the filtering error covariance matrix ${\hat{\Sigma }}_{k/k}$.*

*The filter ${\hat{x}}_{k/k}$, the innovations $\mu_{k+h}$, their covariance matrices ${\hat{\Sigma }}_{k/k}$ and $\Pi_{k+h}$, and the matrices $E_{k+h}$ and $\Sigma_{k}^{e}$ were obtained from Theorem 1.*

**Proof.**  See Appendix B. ☐

## 4. Numerical Simulation Example

The performance of the proposed centralized filtering and fixed-point smoothing algorithms was analyzed in a numerical simulation example which also shows how some of the sensor uncertainties covered by the current measurement model (1) with random parameter matrices influence the accuracy of the estimators. Also, the effect of the random transmission delays and packet losses on the performance of the estimators was analyzed.

### 4.1. Signal Process

Consider a discrete-time scalar signal process generated by the following model with the state-dependent multiplicative noise

$$x_{k+1}=\left(0.9+0.01\varepsilon_{k}\right)x_{k}+w_{k},\;k\geq 0,$$

where $x_{0}$ is a standard Gaussian variable, and ${\left\{w_{k}\right\}}_{k\geq 0},\;{\left\{\varepsilon_{k}\right\}}_{k\geq 0}$ are zero-mean Gaussian white processes with unit variance. Assuming that $x_{0}$, ${\left\{w_{k}\right\}}_{k\geq 0}$ and ${\left\{\varepsilon_{k}\right\}}_{k\geq 0}$ are mutually independent, the signal covariance is given by $E\left[x_{k}x_{s}\right]=0.9^{k-s}D_{s},\;s\leq k,$ where $D_{s}=E\left[x_{s}^{2}\right]$ is obtained by

$$D_{s}=\left(0.9^{2}+0.01^{2}\right)D_{s-1}+1,\;s\geq 1;\;\;D_{0}=1.$$

Hence, the hypothesis (H1) is satisfied with, for example, $A_{k}=0.9^{k}$ y $B_{s}=0.9^{-s}$ $D_{s}$.

This signal process has been considered in the current authors’ previous papers and shows that hypothesis (H1) regarding the signal autocovariance function is satisfied for uncertain systems with state-dependent multiplicative noise. Also, situations where the system matrix in the state-space model is singular are covered by hypothesis (H1) (see, e.g., ref. [9]). Hence, this hypothesis provides a unified general context to deal with different situations, thus avoiding the derivation of specific algorithms for each of them.

### 4.2. Sensor Measured Outputs

As in ref. [20], let us consider four sensors that provide scalar measurements with different random failures, which are described using random parameters according to the theoretical model (1). Namely, sensor 1 has continuous gain degradation, sensor 2 has discrete gain degradation, sensor 3 has missing measurements and sensor 4 has both missing measurements and multiplicative noise. Specifically, the scalar measured outputs are described according to the model

$$z_{k}^{\left(i\right)}=H_{k}^{\left(i\right)}x_{k}+v_{k}^{\left(i\right)},\;\;\;k\geq 1,\;\;\;\;\;i=1,2,3,4,$$

where the additive noises are defined as $v_{k}^{\left(i\right)}=c_{i}\eta_{k}$, with $c_{1}=1$, $c_{2}=0.5$, $c_{3}=c_{4}=0.75$, and ${\left\{\eta_{k}\right\}}_{k\geq 1}$ is a Gaussian white sequence with a mean of 0 and variance of 0.5. The additive noises are correlated with $R_{k}^{\left(ij\right)}=0.5c_{i}c_{j},$ k≥1;i,j=1,2,3,4. The random measurement matrices are defined by $H_{k}^{\left(i\right)}=\theta_{k}^{\left(i\right)}C_{k}^{\left(i\right)}$, for i=1,2,3, where $C_{k}^{\left(1\right)}=0.82$, $C_{k}^{\left(2\right)}=0.75$, $C_{k}^{\left(3\right)}=0.74$, and $H_{k}^{\left(4\right)}=\theta_{k}^{\left(4\right)}\left(0.75+0.95\varphi_{k}\right)$, where the sequence ${\left\{\varphi_{k}\right\}}_{k\geq 1}$ is a standard Gaussian white process, and ${\left\{\theta_{k}^{\left(i\right)}\right\}}_{k\geq 1},\;i=1,2,3,4$, are also white processes with time-invariant probability distributions that are given as follows:

- ${\left\{\theta_{k}^{\left(1\right)}\right\}}_{k\geq 1}$ are uniformly distributed over [0.2,0.7].
- $P\left(\theta_{k}^{\left(2\right)}=0\right)=0.3,\;\;P\left(\theta_{k}^{\left(2\right)}=0.5\right)=0.3,\;\;P\left(\theta_{k}^{\left(2\right)}=1\right)=0.4,\;\;k\geq 1.$
- For i=3,4, ${\left\{\theta_{k}^{\left(i\right)}\right\}}_{k\geq 1}$ are Bernoulli random variables with $P\left(\theta_{k}^{\left(i\right)}=1\right)={\bar{\theta }}^{\left(i\right)},\;\;k\geq 1$.

### 4.3. Model for the Measurements Processed

Now, according to the theoretical study, we assume that the sensor measurements, $y_{k}$, that are processed to update the estimators are modeled by

$$y_{k}=\left(\begin{array}{c} \left(I_{4}-\Gamma_{k}\right)z_{k}+\Gamma_{k}{\hat{z}}_{k/k-1}\\ \Psi_{k}z_{k-1} \end{array}\right),\;\;k\geq 2;\;\;\;\;y_{1}=\left(\begin{array}{c} \left(I_{4}-\Gamma_{1}\right)z_{1}\\ 0 \end{array}\right),$$

where $\Gamma_{k}=Diag\left(\gamma_{k}^{\left(1\right)},\gamma_{k}^{\left(2\right)},\gamma_{k}^{\left(3\right)},\gamma_{k}^{\left(4\right)}\right)$ and $\Psi_{k}=Diag\left(\psi_{k}^{\left(1\right)},\psi_{k}^{\left(2\right)},\psi_{k}^{\left(3\right)},\psi_{k}^{\left(4\right)}\right)$, and for i=1,2,3,4, ${\left\{\gamma_{k}^{\left(i\right)}\right\}}_{k\geq 1}$ and ${\left\{\psi_{k}^{\left(i\right)}\right\}}_{k\geq 2}$ are sequences of independent Bernoulli random variables whose distributions are determined by the following probabilities:

- ${\bar{\gamma }}^{\left(i\right)}\equiv P\left(\gamma_{k}^{\left(i\right)}=1\right),\;k\geq 1$: probability that the measurement $z_{k}^{\left(i\right)}$ is not received at time *k* because it is delayed or lost.
- ${\bar{\psi }}_{\gamma }^{\left(i\right)}\equiv P\left(\psi_{k}^{\left(i\right)}=1/\gamma_{k-1}^{\left(i\right)}=1\right),\;k\geq 1$: probability that the measurement $z_{k-1}^{\left(i\right)}$ is received at the current time (*k*), conditioned to the fact that it is not received on time.
- ${\bar{\psi }}^{\left(i\right)}\equiv P\left(\psi_{k}^{\left(i\right)}=1\right)={\bar{\psi }}_{\gamma }^{\left(i\right)}{\bar{\gamma }}^{\left(i\right)},\;k\geq 1$: probability that the measurement $z_{k-1}^{\left(i\right)}$ is received and processed at the current time *k*.

Finally, in order to apply the proposed algorithms, it was assumed that all the processes involved in the observation equations satisfy the independence hypotheses imposed on the theoretical model.

To illustrate the feasibility and effectiveness of the proposed algorithms, they were implemented in MATLAB, and fifty iterations of the filtering and fixed-point smoothing algorithms were performed. The estimation accuracy was examined by analyzing the error variances for different probabilities of the Bernoulli variables modeling the random failures in sensors 3 and 4 (${\bar{\theta }}^{\left(i\right)},\;i=3,4$). Also, different values of the probabilities ${\bar{\gamma }}^{\left(i\right)}$, corresponding to the transmission uncertainties, and different conditional probabilities ${\bar{\psi }}_{\gamma }^{\left(i\right)}$, i=1,2,3,4, were considered in order to analyze the effect of these failures on the estimation accuracy.

In the study of the performance of the centralized estimators, they were compared with local ones, which were computed using only the measurements received from each single sensor. In that case, the measurements processed at each local processor can be described by

$$y_{k}^{\left(i\right)}=\left(\begin{array}{c} \left(1-\gamma_{k}^{\left(i\right)}\right)z_{k}+\gamma_{k}^{\left(i\right)}{\hat{z}}_{k/k-1}^{\left(i\right)}\\ \psi_{k}^{\left(i\right)}z_{k-1} \end{array}\right),\;\;k\geq 2;\;\;\;\;y_{1}^{\left(i\right)}=\left(\begin{array}{c} \left(1-\gamma_{1}^{\left(i\right)}\right)z_{1}^{\left(i\right)}\\ 0 \end{array}\right),\;\;\;\;i=1,2,3,4,$$

where ${\hat{z}}_{k/k-1}^{\left(i\right)}$ is the one-stage predictor of $z_{k}^{\left(i\right)}$ based on $y_{1}^{\left(i\right)},\ldots ,y_{k-1}^{\left(i\right)}$, and the corresponding local estimators are obtained via recursive algorithms similar to those in Theorems 1 and 2.

### 4.4. Performance of the Centralized Fusion Filtering and Smoothing Estimators

**Figure 2:**
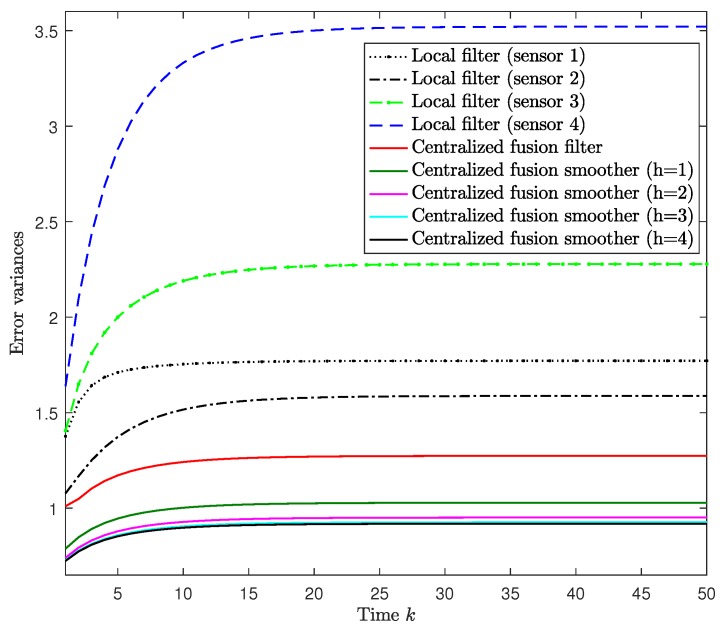
Error variance comparison of the local filters and centralized fusion filter and smoothers.

For i=1,2,3,4, we assumed that ${\bar{\gamma }}^{\left(i\right)}={\bar{\psi }}_{\gamma }^{\left(i\right)}=0.1i$, and that the missing probabilities $1-{\bar{\theta }}^{\left(i\right)}$ had the same value in sensors i=3,4, namely, ${\bar{\theta }}^{\left(i\right)}=0.5$, i=3,4. The error variances of the local filtering estimators and both the centralized filtering and smoothing error variances are displayed in Figure 2. This figure shows, on the one hand, that the error variances of the centralized fusion filtering estimators are significantly smaller than those of every local estimator. Consequently, agreeing with what is theoretically expected, the centralized fusion filter has better accuracy than the local ones, as it is the optimal one based on the information from all the contributing sensors. On the other hand, Figure 2 also shows that as more observations are considered in the estimation, the error variances are lower and consequently, the performance of the centralized estimators becomes better. In other words, the smoothing estimators are more accuracy than the filtering ones, and the accuracy of the smoothers at each fixed-point *k* is better as the number of available observations k+h increases, although this improvement is practically imperceptible for h>3. Similar results were obtained for other values of the probabilities ${\bar{\theta }}^{\left(i\right)}$, ${\bar{\gamma }}^{\left(i\right)}$ and ${\bar{\psi }}_{\gamma }^{\left(i\right)}$.

### 4.5. Influence of the Missing Measurement Phenomenon in Sensors 3 and 4

**Figure 3:**
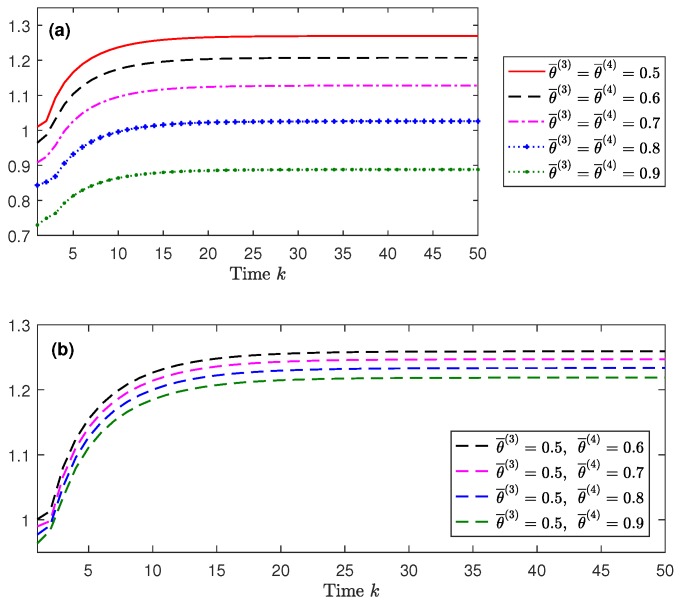
Centralized fusion filtering error variances for different values of ${\bar{\theta }}^{\left(3\right)}$ and ${\bar{\theta }}^{\left(4\right)}$: (**a**) ${\bar{\theta }}^{\left(3\right)}={\bar{\theta }}^{\left(4\right)}$ from 0.5 to 0.9; (**b**) ${\bar{\theta }}^{\left(3\right)}=0.5$ and ${\bar{\theta }}^{\left(4\right)}$ from 0.6 to 0.9.

Considering ${\bar{\gamma }}^{\left(i\right)}={\bar{\psi }}_{\gamma }^{\left(i\right)}=0.1i$, i=1,2,3,4, again, in order to show the effect of the missing probabilities in sensors i=3,4, the centralized filtering error variances are displayed in Figure 3 for different values of these probabilities $1-{\bar{\theta }}^{\left(i\right)}$. Specifically, in Figure 3a, it is assumed that ${\bar{\theta }}^{\left(3\right)}={\bar{\theta }}^{\left(4\right)}$ with a range of values from 0.5 to 0.9, and in Figure 3b, ${\bar{\theta }}^{\left(3\right)}=0.5$ and ${\bar{\theta }}^{\left(4\right)}$ varies from 0.6 to 0.9. From these figures, it is clear that the performance of the centralized fusion filter is indeed influenced by the probabilities ${\bar{\theta }}^{\left(i\right)},\;i=3,4$. Specifically, the performance of the centralized filter is poorer as ${\bar{\theta }}^{\left(i\right)}$ decreases, which means that, as expected, the lower the probability of missing measurements is, the better performance the filter has. Analogous results were obtained for the centralized smoothers and for other values of the probabilities.

Considering that the behavior of the error variances was analogous in all of the iterations, only the results at a specific iteration (k=50) are displayed in the following figures.

### 4.6. Influence of the Probabilities ${\bar{\gamma }}^{\left(i\right)}$ and ${\bar{\psi }}_{\gamma }^{\left(i\right)}$

**Figure 4:**
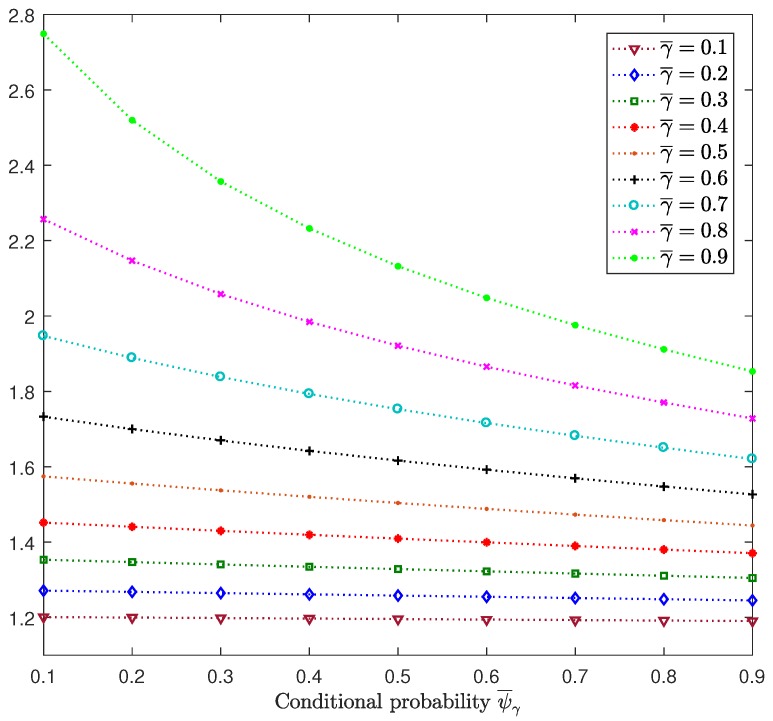
Centralized filtering error variances at k=50 versus ${\bar{\psi }}_{\gamma }$, for $\bar{\gamma }$, varying from 0.1 to 0.9 when ${\bar{\theta }}^{\left(i\right)}=0.5$, i=3,4.

Considering ${\bar{\theta }}^{\left(i\right)}=0.5$, i=3,4, as in Figure 2, we analyze the influences of the random delays and packet dropouts on the performance of the centralized filtering estimators. We assume that the four sensors have the same probability of measurements not arriving on time (${\bar{\gamma }}^{\left(i\right)}=\bar{\gamma },\;i=1,2,3,4$) and also the same conditional probability (${\bar{\psi }}_{\gamma }^{\left(i\right)}={\bar{\psi }}_{\gamma }$, i=1,2,3,4). Figure 4 displays the centralized filtering error variances at k=50 versus ${\bar{\psi }}_{\gamma }$ for $\bar{\gamma }$ varying from 0.1 to 0.9. This figure shows that for each value of $\bar{\gamma }$, the error variances decrease when the conditional probability increases. This result was expected since, for a fixed arbitrary value of $\bar{\gamma }$, the increase in ${\bar{\psi }}_{\gamma }$ entails that of $\bar{\psi }$, which is the probability of processing the delayed measurement at the previous time at the current time. Also, we observed that a decrease in the error variances was more evident for higher values of $\bar{\gamma }$, which was also expected since $\bar{\psi }={\bar{\psi }}_{\gamma }\bar{\gamma }$ and hence, $\bar{\gamma }$ specifies the increasing rate of $\bar{\psi }$ with respect to ${\bar{\psi }}_{\gamma }$.

**Figure 5:**
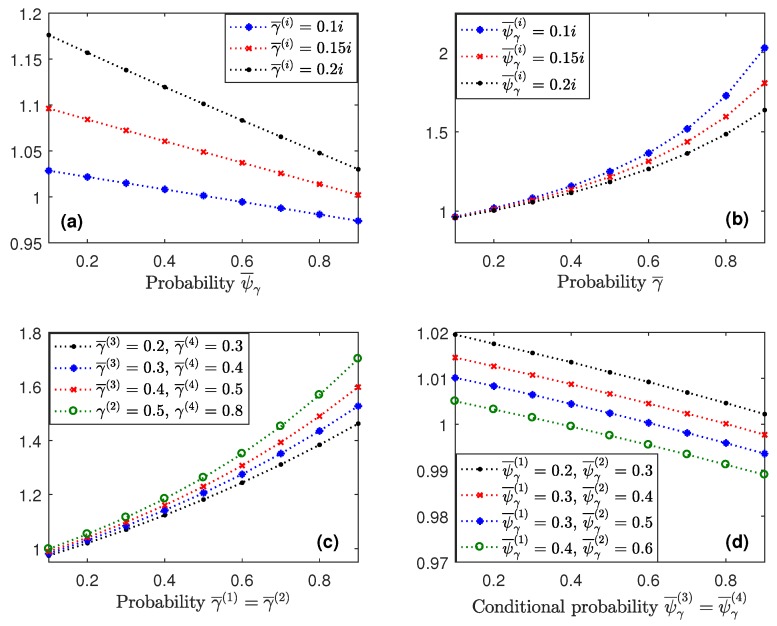
Centralized smoothing error variances (${\hat{\Sigma }}_{50/51}$) when ${\bar{\theta }}^{\left(i\right)}=0.5$ i=3,4, for different values of the probabilities ${\bar{\gamma }}^{\left(i\right)}$ and ${\bar{\psi }}_{\gamma }^{\left(i\right)}$, i=1,2,3,4: (**a**) versus ${\bar{\psi }}_{\gamma }$, for ${\bar{\gamma }}^{\left(i\right)}=0.1i,\;0.15i,\;0.2i$; (**b**) versus $\bar{\gamma }$, for ${\bar{\psi }}_{\gamma }^{\left(i\right)}=0.1i,\;0.15i,\;0.2i$; (**c**) versus ${\bar{\gamma }}^{\left(1\right)}={\bar{\gamma }}^{\left(2\right)}$ for ${\bar{\psi }}_{\gamma }^{\left(i\right)}=0.1i$ and different values of ${\bar{\gamma }}^{\left(3\right)}$ and ${\bar{\gamma }}^{\left(4\right)}$; and (**d**) versus ${\bar{\psi }}_{\gamma }^{\left(3\right)}={\bar{\psi }}_{\gamma }^{\left(4\right)}$, for ${\bar{\gamma }}^{\left(i\right)}=0.1i$ and different values of ${\bar{\psi }}_{\gamma }^{\left(1\right)}$ and ${\bar{\psi }}_{\gamma }^{\left(2\right)}$.

Similar results to the previous ones and consequently, analogous conclusions, were deduced for the smoothing estimators and for different values of the probabilities ${\bar{\gamma }}^{\left(i\right)}$ and ${\bar{\psi }}_{\gamma }^{\left(i\right)}$ at each sensor. By way of example, the smoothing error variances ${\hat{\Sigma }}_{50/51}$ are displayed in Figure 5 for some of the situations considered above.

## 5. Concluding Remarks

In this paper, recursive algorithms were designed for the LS linear centralized fusion prediction, filtering, and smoothing problems in networked systems with random parameter matrices in the measured outputs. At each sampling time, every sensor sends its measured output to the fusion centre where the data packets coming from all the sensors are gathered. Every data packet is assumed to be transmitted just once, but random delays and packet dropouts can occur during this transmission, so the estimator may receive either one packet, two packets, or nothing. When the current measurement of a sensor does not arrive punctually, the corresponding component of the stacked measured output predictor is used as the compensator in the design of the estimators. 

Some of the main advantages of the current approach are the following ones:

- The consideration of random measurement matrices provides a general framework to address different uncertainties, such as missing measurements, multiplicative noise, or sensor gain degradation, as has been illustrated by a simulation example.
- The covariance-based approach used to design the estimation algorithms does not require the knowledge of the state-space model, even though it is also applicable to the classical formulation using this model.
- In contrast to most estimation algorithms dealing with random delays and packet dropouts in the literature, the proposed ones do not require any state vector augmentation technique, and thus are computationally more simple.
- The current estimation algorithms were designed using the LS optimality criterion by a innovation approach and no particular structure of the estimators is required.

## Figures and Tables

**Table 1:** Measurements processed to update the estimators.

Timek	1	2	3	4	5	6	7	8	9	10
$\gamma_{k}$	0	1	0	1	1	0	0	1	0	1
$\psi_{k}$		0	1	0	1	0	0	0	1	0
$y_{k}$	$\left(\begin{array}{c} \;\;\;z_{1}\\ \;\;\;0 \end{array}\;\;\;\right)$	$\left(\begin{array}{c} \;\;\;{\hat{z}}_{1/0}\\ \;\;\;0 \end{array}\;\;\;\right)$	$\left(\begin{array}{c} \;\;\;z_{3}\\ \;\;\;z_{2} \end{array}\;\;\;\right)$	$\left(\begin{array}{c} \;\;\;{\hat{z}}_{4/3}\\ \;\;\;0 \end{array}\;\;\;\right)$	$\left(\begin{array}{c} \;\;\;{\hat{z}}_{5/4}\\ \;\;\;z_{4} \end{array}\;\;\;\right)$	$\left(\begin{array}{c} \;\;\;z_{6}\\ \;\;\;0 \end{array}\;\;\;\right)$	$\left(\begin{array}{c} \;\;\;z_{7}\\ \;\;\;0 \end{array}\;\;\;\right)$	$\left(\begin{array}{c} \;\;\;{\hat{z}}_{8/7}\\ \;\;\;0 \end{array}\;\;\;\right)$	$\left(\begin{array}{c} \;\;\;z_{9}\\ \;\;\;z_{8} \end{array}\;\;\;\right)$	$\left(\begin{array}{c} \;\;\;{\hat{z}}_{10/9}\\ \;\;\;0 \end{array}\;\;\;\right)$

## Appendix A

**Proof** **of** **Theorem** **1.** Based on the general expression (8), to obtain the LS linear estimators ${\hat{x}}_{k/L}$, L≤k, it is necessary to calculate the coefficients

$$X_{k,h}\equiv E\left[x_{k}\mu_{h}^{T}\right]=E\left[x_{k}y_{h}^{T}\right]-E\left[x_{k}{\hat{y}}_{h/h-1}^{T}\right],\;\;h\leq k.$$

Using (5) for $y_{h}$, the independence hypotheses and the factorization of the signal covariance (H1) lead to $E\left[x_{k}y_{h}^{T}\right]=A_{k}{\bar{H}}_{B_{h}}^{T}+E\left[x_{k}{\hat{x}}_{h/h-1}^{T}\right]{\bar{H}}_{h}^{T}{\bar{\Gamma }}_{h}C_{0}^{T},\;\;2\leq h\leq k$, and $E\left[x_{k}y_{1}^{T}\right]=A_{k}{\bar{H}}_{B_{1}}^{T}$, with ${\bar{H}}_{B_{h}}$ given in (15). Now, using expression (10) for ${\hat{y}}_{h/h-1},$ together with (8) for ${\hat{x}}_{h/h-1}$ and ${\hat{x}}_{h-1/h-1}$, the coefficients $X_{k,h}$, 1≤h≤k, are expressed as follows:

$$\begin{array}{c} X_{k,h}=A_{k}{\bar{H}}_{B_{h}}^{T}-\sum_{j=1}^{h-1}X_{k,j}\Pi_{j}^{-1}\left(X_{h,j}^{T}{\bar{H}}_{h}^{T}\left(I_{mn_{z}}-{\bar{\Gamma }}_{h}\right)C_{0}^{T}+X_{h-1,j}^{T}{\bar{H}}_{h-1}^{T}{\bar{\Psi }}_{h}C_{1}^{T}\right)-X_{k,h-1}\Pi_{h-1}^{-1}V_{h-1}^{T},\;\;2\leq h\leq k;\\ X_{k,1}=A_{k}{\bar{H}}_{B_{1}}^{T}, \end{array}$$

which guarantees that $X_{k,h}=A_{k}E_{h},\;1\leq h\leq k$, with $E_{h}$ given by

$$E_{h}={\bar{H}}_{B_{h}}^{T}-\sum_{j=1}^{h-1}E_{j}\Pi_{j}^{-1}E_{j}H_{A_{h}}^{T}-E_{h-1}\Pi_{h-1}^{-1}V_{h-1}^{T},\;\;h\geq 2;\;\;\;\;E_{1}={\bar{H}}_{B_{1}}^{T}.$$

Then, by defining $e_{L}\equiv \sum_{h=1}^{L}E_{h}\Pi_{h}^{-1}\mu_{h}$ and $\Sigma_{L}^{e}\equiv E\left[e_{L}e_{L}^{T}\right]=\sum_{h=1}^{L}E_{h}\Pi_{h}^{-1}E_{h}$, for L≥1, and taking into account that $E\left[e_{L}\mu_{h}^{T}\right]=E_{h}$, for h≤L, it is easy to obtain expressions (11)–(16). Next, the expression (17) for $V_{L}=E\left[V_{L}\mu_{L}^{T}\right]=E\left[V_{L}y_{L}^{T}\right]$ is derived. Using (9) for $V_{L}$, we write $V_{L}=C_{1}\left(E\left[\Psi_{L+1}z_{L}y_{L}^{T}\right]-{\bar{\Psi }}_{L+1}{\bar{H}}_{L}E\left[x_{L}y_{L}^{T}\right]\right)$, and we calculate each of these expectations:

- From (5), we write $y_{L}=C_{0}\left(I_{mn_{z}}-\Gamma_{L}\right)\left(z_{L}-{\hat{z}}_{L/L-1}\right)+C_{1}\Psi_{L}z_{L-1}+C_{0}{\hat{z}}_{L/L-1}$, and from the independence properties, it is clear that
  $$\begin{array}{cc} E\left[\Psi_{L+1}z_{L}y_{L}^{T}\right] & =E\left[\Psi_{k+1}z_{L}{\left(z_{L}-{\hat{z}}_{L/L-1}\right)}^{T}\left(I_{mn_{z}}-\Gamma_{L}\right)\right]C_{0}^{T}\\ & +{\bar{\Psi }}_{L+1}E\left[z_{L}z_{L-1}^{T}\right]{\bar{\Psi }}_{L}C_{1}^{T}+{\bar{\Psi }}_{L+1}E\left[z_{L}{\hat{z}}_{L/L-1}^{T}\right]C_{0}^{T}. \end{array}$$
  Now, from the Hadamard product properties, we obtain $E\left[\Psi_{L+1}z_{L}{\left(z_{L}-{\hat{z}}_{L/L-1}\right)}^{T}\left(I_{mn_{z}}-\Gamma_{L}\right)\right]=(K_{L+1,L}^{\psi (1-\gamma )}\circ \left(\Sigma_{L}^{z}-E{\left[z_{L}{\hat{z}}_{L/L-1}\right]}^{T}\right)$; from property (P5), $E\left[z_{L}z_{L-1}^{T}\right]={\bar{H}}_{L}A_{L}B_{L-1}^{T}{\bar{H}}_{L-1}^{T}$, and using the OPL and (2), $E\left[z_{L}{\hat{z}}_{L/L-1}^{T}\right]=E\left[{\hat{z}}_{L/L-1}{\hat{z}}_{L/L-1}^{T}\right]={\bar{H}}_{L}E\left[{\hat{x}}_{L/L-1}{\hat{x}}_{L/L-1}^{T}\right]{\bar{H}}_{L}^{T}$. Then, using (11) and the definition of $\Sigma_{L}^{e}$, the following expression is obtained:
  $$\begin{array}{cc} E\left[\Psi_{L+1}z_{L}y_{L}^{T}\right] & =\left(K_{L+1,L}^{\psi (1-\gamma )}\circ \left(\Sigma_{L}^{z}-{\bar{H}}_{L}A_{L}\Sigma_{L-1}^{e}A_{L}^{T}{\bar{H}}_{L}^{T}\right)\right)C_{0}^{T}\\ & +{\bar{\Psi }}_{L+1}{\bar{H}}_{L}A_{L}\left(B_{L-1}^{T}{\bar{H}}_{L-1}^{T}{\bar{\Psi }}_{L}C_{1}^{T}+\Sigma_{L-1}^{e}A_{L}^{T}{\bar{H}}_{L}^{T}C_{0}^{T}\right). \end{array}$$
- Using (2), (5) again and the OPL, together with Hypothesis (H1) and (15) for ${\bar{H}}_{B_{L}}$, we have
  $${\bar{\Psi }}_{L+1}{\bar{H}}_{L}E\left[x_{L}y_{L}^{T}\right]={\bar{\Psi }}_{L+1}{\bar{H}}_{L}A_{L}\left({\bar{H}}_{B_{L}}^{T}+\Sigma_{L-1}^{e}A_{L}^{T}{\bar{H}}_{L}^{T}{\bar{\Gamma }}_{L}C_{0}^{T}\right).$$

From the above items and using (15), $B_{L-1}^{T}{\bar{H}}_{L-1}^{T}{\bar{\Psi }}_{L}C_{1}^{T}-{\bar{H}}_{B_{L}}^{T}=-B_{L}^{T}{\bar{H}}_{L}^{T}\left(I_{mn_{z}}-{\bar{\Gamma }}_{L}\right)C_{0}^{T},$ expression (17) is deduced with no difficulty. To obtain expression (18) for $\Pi_{L}=E\left[\mu_{L}\mu_{L}^{T}\right]$, we apply the OPL to write $\Pi_{L}=E\left[y_{L}y_{L}^{T}\right]-E\left[{\hat{y}}_{L/L-1}{\hat{y}}_{L/L-1}^{T}\right]$. On the one hand, using the OPL again, we express $E\left[{\hat{y}}_{L/L-1}{\hat{y}}_{L/L-1}^{T}\right]=E\left[{\hat{y}}_{L/L-1}y_{L}^{T}\right]$ which, takes (16) into account for ${\hat{y}}_{L/L-1}$, and the definitions of $O_{L,L-1}$ and $Y_{L,L-1}$, clearly satisfy

$$E\left[{\hat{y}}_{L/L-1}y_{L}^{T}\right]=\left({\bar{H}}_{A_{L}}+C_{0}{\bar{\Gamma }}_{L}{\bar{H}}_{L}A_{L}\right)O_{L,L-1}^{T}+V_{L-1}\Pi_{L-1}^{-1}Y_{L,L-1}^{T},\;\;L\geq 2.$$

On the other hand, to obtain $E\left[y_{L}y_{L}^{T}\right]$, we use (9) and (6) to write $y_{L}=\xi_{L}+C_{0}\Gamma_{L}{\hat{z}}_{L/L-1}$, and since ${\hat{z}}_{L/L-1}={\bar{H}}_{L}A_{L}e_{L-1}$, the following expression is obtained from the definition of $\Sigma_{L}^{e}$ after some manipulations:

$$\begin{array}{c} E\left[y_{L}y_{L}^{T}\right]\;=\Sigma_{L}^{\xi^{}}-C_{0}\left[K_{L}^{\gamma }\circ \left({\bar{H}}_{L}A_{L}\Sigma_{L-1}^{e}A_{L}^{T}{\bar{H}}_{L}^{T}\right)\right]C_{0}^{T}+C_{0}{\bar{\Gamma }}_{L}{\bar{H}}_{L}A_{L}O_{L,L-1}^{T}+O_{L,L-1}A_{L}^{T}{\bar{H}}_{L}^{T}{\bar{\Gamma }}_{L}C_{0}^{T},\;\;L\geq 2. \end{array}$$

From the above expectations, again, after some manipulations, expression (18) for $\Pi_{L}$ is obtained. To complete the proof, expression (19) for $O_{L,L-1}=E\left[y_{L}e_{L-1}^{T}\right]$ and $Y_{L,L-1}=E\left[y_{L}\mu_{L-1}^{T}\right]$ is derived. Using the OPL, we have $E\left[y_{L}e_{L-1}^{T}\right]=E\left[{\hat{y}}_{L/L-1}e_{L-1}^{T}\right]$ and $E\left[y_{L}\mu_{L-1}^{T}\right]=E\left[{\hat{y}}_{L/L-1}\mu_{L-1}^{T}\right]$, and from (16) for ${\hat{y}}_{L/L-1}$, expression (19) is straightforward. Then, the proof of Theorem 1 is complete.

## Appendix B

**Proof** **of** **Theorem** **2.** Using (8), the signal estimators are written as ${\hat{x}}_{k/k+h}=\sum_{l=1}^{k+h}X_{k,l}\Pi_{l}^{-1}\mu_{l},\;h\geq 1$, from which it is immediately deduced that the smoothers are recursively obtained by (20) from the filter ${\hat{x}}_{k/k}$. Taking into account that $X_{k,k+h}=E\left[x_{k}y_{k+h}^{T}\right]-E\left[x_{k}{\hat{y}}_{k+h/k+h-1}^{T}\right],\;h\geq 1$, the recursive relation (21) is derived by just calculating each of these expectations as follows:

- Hypothesis (H1) together with (15), leads to
  $$E\left[x_{k}y_{k+h}^{T}\right]=B_{k}{\bar{H}}_{A_{k+h}}^{T}+E\left[x_{k}e_{k+h-1}^{T}\right]A_{k+h}^{T}{\bar{H}}_{k+h}^{T}{\bar{\Gamma }}_{k+h}C_{0}^{T},\;\;h\geq 1.$$
- From (16) for ${\hat{y}}_{k+h/k+h-1}$, it is clear that
  $$\begin{array}{c} E\left[x_{k}{\hat{y}}_{k+h/k+h-1}^{T}\right]=E\left[x_{k}e_{k+h-1}^{T}\right]{\left({\bar{H}}_{A_{k+h}}+C_{0}{\bar{\Gamma }}_{k+h}{\bar{H}}_{k+h}A_{k+h}\right)}^{T}+X_{k,k+h-1}\Pi_{k+h-1}^{-1}V_{k+h-1}^{T},\;\;h\geq 1. \end{array}$$

From the above items, (21) is proven simply by denoting $E_{k,k+h}=E\left[x_{k}e_{k+h}^{T}\right]$, whose recursive expression (22) is also obvious by using (12) for $e_{k+h}$. Finally, using (20) for the smoothers ${\hat{x}}_{k/k+h}$, the recursive formula for the fixed-point smoothing error covariance matrices ${\hat{\Sigma }}_{k/k+h}$ is immediately deduced. ☐
